# Pharmacokinetics of Maleic Acid as a Food Adulterant Determined by Microdialysis in Rat Blood and Kidney Cortex

**DOI:** 10.3390/molecules21030367

**Published:** 2016-03-17

**Authors:** Mei-Ling Hou, Chia-Ming Lu, Chi-Hung Lin, Lie-Chwen Lin, Tung-Hu Tsai

**Affiliations:** 1 Institute of Traditional Medicine, School of Medicine, National Yang-Ming University, No. 155, Section 2, Li-Nong Street, Beitou District, Taipei 11221, Taiwan; maylinghou@gmail.com (M.-L.H.); a121060@gmail.com (C.-M.L.); 2 Institute of Microbiology and Immunology, National Yang-Ming University, No. 155, Section 2, Li-Nong Street, Beitou District, Taipei 11221, Taiwan; linch@ym.edu.tw; 3 National Research Institute of Chinese Medicine, Ministry of Health and Welfare, No. 155-1, Section 2, Li-Nong Street, Beitou District, Taipei 11221, Taiwan; lclin@nricm.edu.tw; 4 Graduate Institute of Acupuncture Science, China Medical University, No. 91, Hsueh-Shih Road, Taichung 40402, Taiwan; 5 School of Pharmacy, College of Pharmacy, Kaohsiung Medical University, 100, Shih-Chuan 1st Road, Kaohsiung 80708, Taiwan; 6 Department of Education and Research, Taipei City Hospital, No. 145, Zhengzhou Road, Datong District, Taipei 103, Taiwan

**Keywords:** maleic acid, food adulterant, pharmacokinetics, microdialysis, liquid chromatography, kidney distribution

## Abstract

Maleic acid has been shown to be used as a food adulterant in the production of modified starch by the Taiwan Food and Drug Administration. Due to the potential toxicity of maleic acid to the kidneys, this study aimed to develop an analytical method to investigate the pharmacokinetics of maleic acid in rat blood and kidney cortex. Multiple microdialysis probes were simultaneously inserted into the jugular vein and the kidney cortex for sampling after maleic acid administration (10 or 30 mg/kg, i.v., respectively). The pharmacokinetic results demonstrated that maleic acid produced a linear pharmacokinetic phenomenon within the doses of 10 and 30 mg/kg. The area under concentration *versus* time curve (AUC) of the maleic acid in kidney cortex was 5-fold higher than that in the blood after maleic acid administration (10 and 30 mg/kg, i.v., respectively), indicating that greater accumulation of maleic acid occurred in the rat kidney.

## 1. Introduction

Maleic acid and maleic anhydride are multi-functional chemical intermediates with many industrial applications such as a fragrance ingredient, pH adjuster in cosmetics, and food contact materials. However, its use as a food additive is banned in both Europe and the US [1]. As a pH adjuster, maleic acid is used in a few cosmetic product formulations at low concentrations. Maleic acid is used in manufacturing artificial resins, dyeing and finishing wools, cottons, and silks; and in antihistamine salts. It may also be used as an adhesive on articles intended for use in packing, transporting, or holding food [1]. The Taiwan Food and Drug Administration recently found that maleic acid was being used as an adulterant in starch without approval for the production of modified starch.

Maleic acid’s mode of action has been determined in general biology and animal toxicology [1,2,3,4]. In terms of genotoxicity, maleic acid failed to induce any significant increases in the revertant count in any of the tested strains [3]. However, maleic acid showed a positive pattern in the DNA synthesis inhibition test; specifically, the rate of DNA synthesis at 90 min was greatly suppressed. The ocular safety of excipient maleic acid following intravitreal injection was assessed in rabbits [2]; the data suggest that maleic acid is toxic to the eyes of rabbits in a concentration-dependent manner. Maleic acid is also a nephrotoxin, causing glycosuria, phosphaturia, and aminoaciduria [5,6,7]. The renal glycosuria, phosphaturia, and aminoaciduria induced by maleic acid are similar to a congenital defect in humans known as Fanconi syndrome, a generalized proximal tubular reabsorptive dysfunction. Consequently, maleic acid is commonly used to induce Fanconi syndrome in rats [5,8], mice [6] and dogs [4] to study the mechanism of the disease. Maleic acid at 9 mmol/kg decreased the glomerular filtration rate in rats, in association with inhibition of Na^+^/K^+^-ATPases activity in the proximal tubule.

Analytical methods have been reported for determination of maleic acid in general foods, in fruits and fruit juice using capillary electrophoresis (CE) [9], ion pair liquid chromatography [10], liquid chromatography with ultraviolet (UV) detection [11,12], liquid chromatography with electrospray ionization tandem mass spectrometry [13], and capillary electrophoresis-electrospray ionization ion-trap mass spectrometry [14]. The capability and reliability of the microdialysis technique for measuring endogenous substances as well as exogenous therapeutic agents in various tissue systems have brought it to the forefront of the *in vivo* tissue sampling methods [15]. This technique has been widely used for continuous analysis of different substances contained in different organs such as the kidney cortex [16]. The advantages of the microdialysis technique include not only simultaneously sampling at multiple sites but also the fact that no sample preparation is necessary as the dialysis membrane excludes proteins from the aqueous sample.

To date, the information on pharmacokinetics of maleic acid is limited; no pharmacokinetic- or physiologically-based pharmacokinetic models have been investigated for maleic acid in rats using microdialysis techniques. Since maleic acid was used illegally to modify starch as food additives, the pharmacokinetics of maleic acid in rat blood and renal cortex were investigated to elucidate the profiles after maleic acid administration. Due to the potential toxicity of maleic acid to the kidneys, the aim of the present study is to develop an analytical method to simultaneously investigate the pharmacokinetics of maleic acid in rat blood and kidney dialysates using microdialysis sampling combined with an HPLC-photodiode array (PDA) detector method. The AUC ratio (AUC_kidney_/AUC_blood_) in the kidneys and the blood was used to define the distribution of kidney-to-blood maleic acid.

## 2. Results

### 2.1. HPLC Method Validation

**Figure 1:**
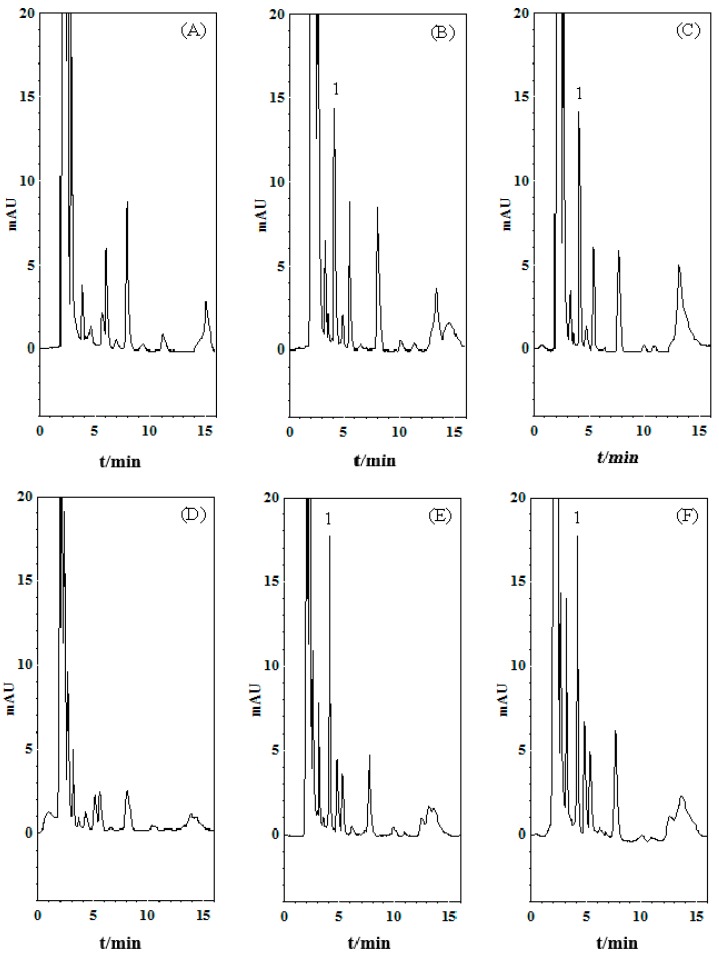
Representative HPLC chromatograms of (**A**) blank blood dialysate; (**B**) blank blood dialysate spiked with maleic acid (1 μg/mL); (**C**) blood dialysate sample containing maleic acid (0.98 μg/mL) collected at 60–75 min after maleic acid administration (30 mg/kg, i.v.); (**D**) blank kidney dialysate; (**E**) blank kidney dialysate spiked with maleic acid (1 μg/mL) and (**F**) kidney dialysate sample containing maleic acid (1.20 μg/mL) collected at 105–120 min after maleic acid administration (30 mg/kg, i.v.). 1: maleic acid.

The HPLC-PDA analytical method was used to determine maleic acid concentrations in the rat blood and kidney dialysates. As shown in Figure 1, there was a small amount of endogenous interference in the blank rat blood and kidney dialysate samples. However, the endogenous interference did not influence the maleic acid quantification. The selectivity was tested by chromatography of the blank dialysate samples spiked with the maleic acid standards. Good linearity was achieved over the range of 0.5–25 µg/mL, with all of the coefficients of correlation greater than 0.995. Under the present analytical method, the maleic acid retention time was 4.1 min.

Intra- and inter-day precision (% RSD) and accuracy (% Bias) were determined by repeated analyses of the six lots of blank dialysate samples spiked with varying maleic acid concentrations on the same day and over six consecutive days, respectively. The precision and accuracy results are presented in Table 1. Intra- and inter-day precision ranged from 0.05% to 7.75%, and accuracy ranged from −4.21% to 3.06% in the rat blood dialysate samples. In addition, intra- and inter-day precision ranged from 0.16% to 6.89%, and accuracy ranged from −0.73% to 1.90% in the rat kidney dialysate samples. The accuracy and precision of the different concentrations were all acceptable. The limit of detection (LOD) and quantification (LOQ) of maleic acid in the rat blood and kidney dialysate samples were 0.1 and 0.5 µg/mL, respectively.

### 2.2. In Vivo Microdialysis Recovery

The average values from the *in vivo* microdialysis recovery of the blood and kidney probes for maleic acid at low (0.5 µg/mL), medium (5 µg/mL) and high (10 µg/mL) concentrations are shown in Table 2. The data showed that there were no significant differences in the recovery of the blood and kidney microdialysis probes at the three concentrations of maleic acid examined. The results appear to suggest that the recovery of the microdialysis probes was independent of the maleic acid concentration.

### 2.3. Blood Pharmacokinetics of Maleic Acid

**Figure 2:**
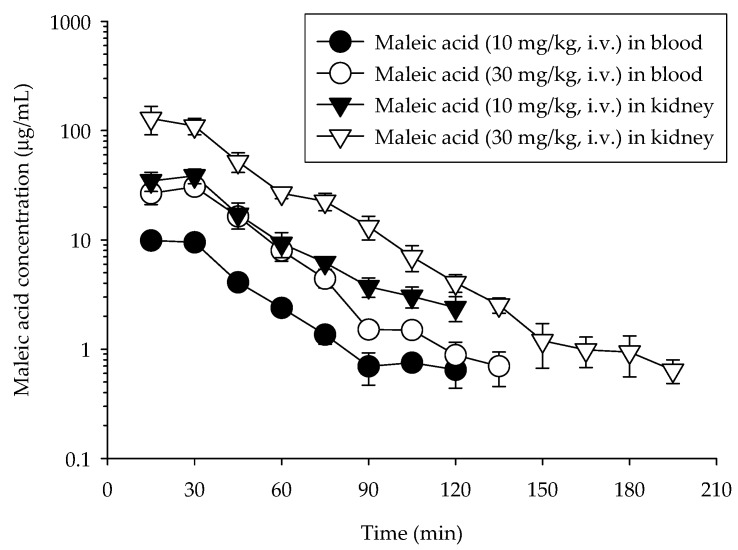
Concentration–time curve of protein-unbound maleic acid in rat blood and kidney after maleic acid administration (10 and 30 mg/kg, i.v., respectively). Data are expressed as mean ± S.E.M. (*n* = 5).

The concentration *versus* time profile of maleic acid in the rat blood dialysate samples after intravenous administration of maleic acid (10 and 30 mg/kg, respectively) to five individual rats for each group are illustrated in Figure 2, and the calculated pharmacokinetic parameters are listed in Table 3. The pharmacokinetic models (one- or two-compartment) were compared according to the Akaike Information Criterion (AIC) values, with the minimum AIC values being regarded as the best representation for the plasma concentration-time course data. The WinNonlin program proposed a compartment model with individual animal data following doses. The average AIC value decreased from 25 for two-compartment model to 18 for the one-compartment model, indicating that the one-compartment model is more suitable than the two-compartment model for the maleic acid administration (Table 3).

The maleic acid level in the blood declined below the LOQ after 120 min. The C_max_ for maleic acid was 10.9 ± 1.08 µg/mL after the administration of 10 mg/kg of maleic acid, and was 32.1 ± 5.17 µg/mL after the administration of 30 mg/kg of maleic acid, reflecting a linear relationship for the maleic acid concentration in the blood. Furthermore, according to the AUC dose ratio, the results demonstrate that the pharmacokinetics of maleic acid is linear. The T_1/2_ of maleic acid in blood was 24.1 min, indicating that the elimination of maleic acid was quick.

### 2.4. Kidney Pharmacokinetics of Maleic Acid

The concentration *versus* time profile of maleic acid in the rat kidney dialysate samples are shown in Figure 2 and the calculated pharmacokinetic parameters are presented in Table 3. Following administration of maleic acid at 10 mg/kg, i.v., the concentrations of maleic acid in the kidney declined below the LOQ after 135 min. With maleic acid at 30 mg/kg, i.v., the maleic acid levels in kidney remained detectable until 195 min. The C_max_ for maleic acid in the kidney was four times higher than in the blood, with a C_max_ of 41.4 ± 5.04 µg/mL after dosing with maleic acid at 10 mg/kg. Furthermore, the T_1/2_ was close to 1.3 times as long as in the blood (T_1/2_ = 32.1 min). The AUC ratio of maleic acid (AUC_kidney_/AUC_blood_) in the kidney and in the blood was defined as the distribution of kidney-to-blood. At a dosage of 10 mg/kg of maleic acid i.v., the AUC in kidney was 5-fold higher than that in blood. Maleic acid at a concentration of 30 mg/kg intravenously yielded similar results. The results demonstrate that kidney has a greater distribution rate and that the pharmacokinetics of maleic acid is linear.

## 3. Discussion

Chromatographic methods such as high-performance liquid chromatography (HPLC) [11,12], and ion chromatography (IC) [10] have been used for the determination of maleic acid in a wide variety of samples. Among them, HPLC and IC are the most commonly used. In HPLC methods, additional sample pretreatment procedures are often required. However, in the microdialysis sampling technique, the obtained dialysate samples can be directly analyzed by the HPLC-PDA method without further sample preparation. The intra- and inter-day precision (% RSD) and accuracy (% bias) strongly indicated that the analytical method was reliable.

The advantage of the microdialysis technique is that only the protein-unbound form of analytes can be sampling. The dialysis efficiency can be affected by factors such as the length, diameter and materials of the membrane; the diffusion coefficient of the analyte; time; temperature; the composition of the perfusion solution; the perfusion flow rate; and the properties of the substances [15,17,18,19,20]. Therefore, the recovery of each probe must be evaluated at the end of the *in vivo* experiment. Although different types of microdialysis probes (home-made and commercial linear probes) and perfusion solutions (ACD and Ringer’s solutions) were used in this study, the mean *in vivo* recovery of maleic acid in the blood and kidney was 33.8% ± 3.95% and 30.7% ± 4.51%, respectively. There were no significant differences on the levels of recovery between the two different probe types.

In contrast with other sampling methods, microdialysis enables continuous sampling of low molecular weight compounds with minimal perturbation in various organs *in vivo* [15,20]. This *in vivo* sampling method continuously measures endogenous substances, as well as drugs and/or their metabolite concentrations, in the extracellular fluid of a specific tissue of freely moving animals or in animals that are restrained or under anesthesia. Regarding the selection of administered routes, maleic acid is used as food adulterant; however, the peristalsis of the gastrointestinal tract will be limited when animals are under anesthesia. Thus, we chose intravenous route instead. As a result, we attempted to study the unbound maleic acid *in vivo* by taking advantage of the microdialysis technique, which reduces the animal requirement and causes less tissue damage, no biological fluid loss, and is sufficiently sensitive to measure the unbound maleic acid in the microdialysates.

The pharmacokinetic data demonstrated that the AUC of maleic acid in blood and kidney were 502 ± 53.4 and 1997 ± 355 min μg/mL, respectively after maleic acid administration (10 mg/kg, i.v.), indicating that maleic acid accumulated higher amounts in the rat kidney. The volume of distribution (V_d_) of maleic acid at 10 and 30 mg/kg, i.v. in the blood are 975 ± 113 and 1050 ± 191 mL/kg, respectively. Additionally, the V_d_ of maleic acid at 10 and 30 mg/kg in the kidney are 209 ± 36.5 and 182 ± 41.9 mL/kg, respectively. The total body clearance (CL) of maleic acid at 10 and 30 mg/kg in the blood are 27.4 ± 2.38 and 25.3 ± 2.54 mL/min/kg, respectively. Moreover, the CL of maleic acid at 10 and 30 mg/kg in the kidney are 5.45 ± 1.06 and 5.34 ± 1.15 mL/min/kg, respectively. These results indicate that V_d_ and CL in the blood are greater than that in the kidney. Since maleic acid was used illegally to modify starch as food additives, the pharmacokinetics of maleic acid in rat blood and renal cortex were investigated to elucidate the profiles after maleic acid administration. Maleic acid is a known nephrotoxicant. Concurrent exposure to subtoxic doses of maleic acid and its chlorinated derivative dichloromaleic acid (DCMA) in both sexes of Sprague-Dawley rats can alter renal function [21]. Female rats appeared to show an enhanced susceptibility to the nephrotoxic action of maleic acid and DCMA in combination. Furthermore, it is well documented that Fanconi syndrome can be induced by maleic acid, which decreases the glomerular filtration rate in association with impairment of the proximal tubule [5,22]. In our pharmacokinetic study, the C_max_ in the kidney was around four times higher than that in the blood, and the AUC in the kidney was five times higher than that in the blood; these results indicate an accumulation of maleic acid in the kidney. Therefore, maleic acid may result in increased risk of nephrotoxicity in humans when used as a food adulterant.

## 4. Experimental Section

### 4.1. Chemicals and Reagents

Maleic acid and urethane were purchased from Sigma-Aldrich Chemicals (St. Louis, MO, USA). HPLC-grade methanol, citric acid, sodium citrate, dextrose, sodium chloride, sodium dihydrogen phosphate (NaH_2_PO_4_), orthophosphoric acid (H_3_PO_4_, 85%) and sodium hydroxide were purchased from E. Merck (Darmstadt, Germany). Triply deionized water (Millipore, Bedford, MA, USA) was used for all of the preparations.

### 4.2. Experimental Animals

All of the animal experimental protocols were reviewed and approved by the Institutional Animal Care and Use Committee (IACUC number: 1020818) of the National Yang-Ming University. Male, pathogen-free Sprague-Dawley rats weighing 220 ± 20 g were obtained from the Laboratory Animal Center of the National Yang-Ming University, Taipei, Taiwan. The animals had free access to food (laboratory rodent diet 5P14, PMI Feeds, Richmond, IN, USA) and water.

### 4.3. HPLC Instrumentation

An HPLC-PDA system (Shimadzu, Kyoto, Japan), equipped with a chromatographic pump (LC-20AT), an autosampler (SIL-20AC), a DGU-20A5 degasser, and a photodiode array detector (SPD-M20A), was used for analysis of maleic acid. Separation was achieved on a reverse-phase C18 column (Purospher STAR, 250 mm × 4 mm, i.d.; particle size of 5 μm, Merck) with an isocratic elution using the mobile phase comprising methanol and 10 mM NaH_2_PO_4_ (1:99, *v*/*v*, adjusted to pH 4.5 using orthophosphoric acid) and a flow rate of 1 mL/min. All of the mobile phase was filtered through a Millipore 0.22 μm filter (EMD Millipore, Bedford, MA, USA) and degassed using a sonicator (Branson, North Olmsted, OH, USA) for 10 min before use. A sample volume of 20 μL was directly injected into the HPLC for analysis without sample preparation. The detection wavelength was set at 210 nm.

### 4.4. Method Validation

The method validation assays for quantification of maleic acid in dialysate samples were carried out according to the currently accepted US Food and Drug Administration (FDA) bioanalytical method validation guidance for specificity, linearity, sensitivity, precision, accuracy and recovery. The specificity was tested by screening six different batches of drug-free blank dialysates for the exclusion of any endogenous co-eluting interference at the peak region of maleic acid. The standard stock solution was prepared by dissolving maleic acid in double-distilled water (ddH_2_O) at a concentration of 1 mg/mL and stored at −20 °C. The stock solution was diluted with ddH_2_O to prepare a series of working standard solutions. The calibration curves were generated by spiking standard solutions (5 μL) in blank rat blood and kidney dialysates (45 μL). The calibration curves were obtained by freshly spiked blood or kidney dialysate with the stock solution of maleic acid at concentration ranges of 0.5–25 µg/mL. All linear curves were required to have a coefficient of estimation of at least more than 0.995. The intra- and inter-day variability were determined by quantitating six replicates at concentrations of 0.5, 1, 5, 10, and 25 µg/mL using the HPLC-PDA method described above on the same day and six consecutive days, respectively. The accuracy (bias %) was calculated from the mean value of observed concentration (C_obs_) and nominal concentration (C_nom_) using the relationship accuracy (bias %) = [(C_obs_ − C_nom_)/C_nom_] × 100. The relative standard deviation (RSD) was calculated from the observed concentrations as precision (RSD %) = (standard deviation (SD)/C_obs_) × 100.

### 4.5. Microdialysis Experiments

The microdialysis system consisted of a CMA400 microinjection pump, a CMA142 microfraction collector (CMA Microdialysis AB, Solna, Sweden) and microdialysis probes. The microdialysis probe for blood sampling was made in our laboratory [23]. The silica capillary was designed for a concentric shape and the tip of the probe was covered with a dialysis membrane (molecular weight cut-off of 13,000 Da, Spectrum, Laguna Hills, CA, USA) 10 mm in length. In addition, the CMA30 linear microdialysis probe (molecular weight cut-off of 6000 Da; membrane of 10 mm in length, CMA Microdialysis AB) was used for the kidney cortex sampling.

The rats were anesthetized intraperitoneally with urethane (1 g/kg). Then, a polyethylene tube (PE-50; BD, Franklin lakes, NJ, USA) was cannulated into the femoral vein for further drug administration. The blood microdialysis probe was positioned in the jugular vein toward the right atrium and perfused with anti-coagulant citrate dextrose (ACD) solution consisting of 3.5 mM citric acid, 7.5 mM sodium citrate, and 13.6 mM dextrose at 2 µL/min. The linear microdialysis probe was implanted in the kidney cortex and perfused with Ringer’s solution consisting of 147 mM sodium chloride, 2.2 mM calcium chloride, and 4 mM potassium chloride at 2 µL/min. After a 2 h post-surgical stabilization period following the implantation of the microdialysis probes, maleic acid (10 or 30 mg/kg) was administered intravenously via the femoral vein cannula. The dialysates from the blood and kidney tissue were collected at 15 min intervals for 3 h into the fraction collector (CMA 402) and analyzed by HPLC-PDA. The dialysates were preserved at −20 °C before analysis.

### 4.6. Recovery Assessment of Microdialysis Probes

A retrodialysis method that measured the loss (extraction ratio) of maleic acid through the probe was utilized to estimate the *in vivo* recoveries [23]. The microdialysis probes were inserted into the jugular vein and the kidney cortex under anesthesia, and the ACD and Ringer’s solutions containing maleic acid at low, medium, and high concentrations (0.5, 5 and 10 μg/mL) were then perfused through the probes at a flow rate of 2 μL/min. The *in vivo* recoveries were evaluated with three individual experiments for each concentration for the blood and kidney microdialysis probes, respectively. The maleic acid perfusate (C_perf_) and dialysate (C_dial_) concentrations were determined by the HPLC-PDA method. The *in vivo* recovery (R_dial_) of maleic acid was calculated by the following equation: R_dial_ = (C_perf_ − C_dial_)/C_perf_. The concentrations of maleic acid were converted to free-form concentrations (C_f_) as follows: C_f_ = C_m_/R_dial_.

### 4.7. Pharmacokinetics

Pharmacokinetic calculations were performed on each individual data set using the pharmacokinetic software WinNonlin Standard Edition, version 1.1 (Pharsight Corp., Mountain View, CA, USA), based on the compartment and non-compartment methods. Akaike’s Information Criterion (AIC) value was applied to select the compartment model used for the data analysis. Based on linear pharmacokinetics, the time course of the plasma concentration (Cp) of a drug was represented as Cp = Ae-αt and Cp = Ae-αt + Be-βt for the one- and two-compartment models, respectively. In the equation, A and B are the concentration (C) intercepts for the fast and slow disposition phases, respectively; α and β are the disposition rate constants for fast and slow disposition phases, respectively. The area under the concentration *versus* time curve (AUC) was calculated according to the log linear trapezoidal method. The drug clearance (Cl) was considered as follows: Cl = dose/AUC. The time required to reduce the drug concentration by half is shown as the half-life (T_1/2_) and was expressed as T_1/2_ = 0.693/K, where K is the first order rate constant. The distribution volume (V_d_) was evaluated as V_d_ = dose/C_0_, where C_0_ is the initial plasma concentration. The mean residence time (MRT) was estimated as MRT = AUMC/AUC, where AUMC is the area under the first moment curve. All of the data are presented as mean ± SEM.

## 5. Conclusions

This is the first study to investigate the pharmacokinetics of the protein-unbound form of maleic acid in rat blood and kidney using continuous microdialysis sampling coupled to an HPLC-PDA method. The method was demonstrated to be selective, precise, accurate, and reliable for the determination of maleic acid concentration in biological dialysate samples. In addition, the results revealed that the AUC of maleic acid in the kidney was 5-fold higher than in the blood and that the pharmacokinetics of maleic acid was linear. The results suggest that maleic acid used as a food adulterant for the production of modified starch may present a potential risk of nephrotoxicity in humans.

## Figures and Tables

**Table 1:** Intra- and inter-day precision (% RSD) and accuracy (% Bias) of the HPLC-PDA method for determination of maleic acid in rat blood and kidney dialysates (6 days, 6 replicates per day).

Nominal Concentration (µg/mL)	Intra-Day			Inter-Day		
	Observed Concentration (µg/mL)	Precision (% RSD)	Accuracy (% Bias)	Observed Concentration (µg/mL)	Precision (% RSD)	Accuracy (% Bias)
Plasma						
0.5	0.48 ± 0.01	1.13	−4.21	0.52 ± 0.04	7.75	3.06
1	0.99 ± 0.01	1.15	−0.83	1.00 ± 0.08	7.70	0.07
5	5.01 ± 0.02	0.41	0.11	5.06 ± 0.08	1.57	1.22
10	10.1 ± 0.04	0.38	0.92	9.88 ± 0.26	2.59	−1.18
25	25.0 ± 0.01	0.05	−0.15	25.0 ± 0.10	0.39	0.11
Kidney						
0.5	0.51 ± 0.03	6.37	1.47	0.51 ± 0.03	5.57	1.90
1	1.02 ± 0.04	3.84	1.83	0.99 ± 0.07	6.89	−0.69
5	4.96 ± 0.04	0.81	−0.73	5.03 ± 0.22	4.43	0.50
10	10.1 ± 0.15	1.47	0.71	9.94 ± 0.27	2.69	−0.62
25	25.0 ± 0.04	0.16	−0.04	25.0 ± 0.09	0.36	0.00

Data expressed as mean ± SD.

**Table 2:** *In vivo* microdialysis recovery (%) of maleic acid in rat blood and kidney.

Concentration (µg/mL)	Recovery (%)
Blood	
0.5	37.7 ± 0.74
5	31.6 ± 0.20
10	32.0 ± 2.12
Average	33.8 ± 3.95
Kidney	
0.5	31.5 ± 1.66
5	31.3 ± 3.08
10	29.8 ± 6.45
Average	30.7 ± 4.51

Data expressed as mean ± SD (*n* = 3).

**Table 3:** Pharmacokinetic parameters of maleic acid after intravenous administration.

Parameters	10 mg/kg, i.v.	30 mg/kg, i.v.
Blood		
AIC of one-compartment	18 ± 2	46 ± 2
AIC of two-compartment	25 ± 3	49 ± 2
AUC (min∙µg/mL)	376 ± 33.4	1236 ± 124
C_max_ (µg/mL)	10.9 ± 1.08	32.1 ± 5.17
T_1/2_ (min)	24.1 ± 1.19	28.4 ± 3.20
CL (mL/min/kg)	27.4 ± 2.38	25.3 ± 2.54
MRT (min)	34.7 ± 1.72	40.9 ± 4.62
V_d_ (mL/kg)	975 ± 113	1050 ± 191
AUC/Dose	37.6	41.2
Kidney		
Non-compartment		
AUC (min∙µg/mL)	1997 ± 355	6784 ± 1538
C_max_ (µg/mL)	41.4 ± 5.04	134 ± 35.6
T_1/2_ (min)	32.1 ± 5.69	22.3 ± 3.18
CL (mL/min/kg)	5.45 ± 1.06	5.34 ± 1.15
MRT (min)	31.8 ± 1.10	32.7 ± 2.16
V_d_ (mL/kg)	209 ± 36.5	182 ± 41.9
AUC/Dose	199.7	226.1
AUC ratio of (AUC_kidney_/AUC_blood_)	5.31	5.49

Data expressed as mean ± S.E.M. (*n* = 5). AUC, area under the concentration *versus* time curve; C_max_, the peak plasma concentration of a drug after administration; T_1/2_, elimination half-life; CL, total body clearance; MRT, mean residence time; V_d_, volume of distribution; the AUC ratio (AUC_kidney_/AUC_blood_) in the kidneys and the blood was used to define the distribution of kidney-to-blood maleic acid.

## Abbreviations

The following abbreviations are used in this manuscript:

AUC	Area under the concentration *versus* time curve
C_max_	The peak plasma concentration of a drug after administration
T_1/2_	Elimination half-life
CL	Total body clearance
MRT	Mean residence time
V_d_	Volume of distribution
CE	Capillary electrophoresis
LOD	The limit of detection
LOQ	The limit of quantification
AIC	Akaike’s information criterion
HPLC-PDA	High performance liquid chromatography photodiode array
IC	Ion chromatography
